# Imaging neural activity in the ventral nerve cord of behaving adult *Drosophila*

**DOI:** 10.1038/s41467-018-06857-z

**Published:** 2018-10-22

**Authors:** Chin-Lin Chen, Laura Hermans, Meera C. Viswanathan, Denis Fortun, Florian Aymanns, Michael Unser, Anthony Cammarato, Michael H. Dickinson, Pavan Ramdya

**Affiliations:** 1 Brain Mind Institute, École Polytechnique Fédérale de Lausanne, CH-1015 Lausanne, Switzerland; 2 Interfaculty Institute of Bioengineering, École Polytechnique Fédérale de Lausanne, CH-1015 Lausanne, Switzerland; 3 Department of Medicine, Johns Hopkins University, Baltimore, MD 21205 USA; 4 Biomedical Imaging Group, École Polytechnique Fédérale de Lausanne, CH-1015 Lausanne, Switzerland; 5 Signal Processing core of the Center for Biomedical Imaging (CIBM-SP), École Polytechnique Fédérale de Lausanne, CH-1015 Lausanne, Switzerland; 6 Department of Physiology, Johns Hopkins University, Baltimore, MD 21205 USA; 7 Biology and Biological Engineering, California Institute of Technology, Pasadena, CA 91125 USA; 8 Present Address: ICube, CNRS, University of Strasbourg, CS 10413 - F-67412 Illkirch Cedex, France

## Abstract

To understand neural circuits that control limbs, one must measure their activity during behavior. Until now this goal has been challenging, because limb premotor and motor circuits have been largely inaccessible for large-scale recordings in intact, moving animals—a constraint that is true for both vertebrate and invertebrate models. Here, we introduce a method for 2-photon functional imaging from the ventral nerve cord (VNC) of behaving adult *Drosophila melanogaster*. We use this method to reveal patterns of activity across nerve cord populations during grooming and walking and to uncover the functional encoding of moonwalker ascending neurons (MANs), moonwalker descending neurons (MDNs), and a previously uncharacterized class of locomotion-associated A1 descending neurons. Finally, we develop a genetic reagent to destroy the indirect flight muscles and to facilitate experimental access to the VNC. Taken together, these approaches enable the direct investigation of circuits associated with complex limb movements.

## Abstract (web summary)

The *Drosophila* ventral nerve cord (VNC) is functionally equivalent to the vertebrate spinal cord. This study reports a 2-photon imaging approach for recording neural activity in the VNC of walking and grooming adult flies.

## Introduction

Limbs allow animals to rapidly navigate complex terrain, groom, manipulate objects, and communicate. In vertebrates, neural circuits in the spinal cord coordinate the actions of each arm or leg. Thoracic circuits perform comparable tasks in insects^1^. The thoracic segments of the fruit fly, *Drosophila melanogaster*, house the ventral nerve cord (VNC) which is a fusion of three thoracic and eight abdominal ganglia. The VNC contains six spherical neuromeres, each controlling one leg, a flat dorsal neuropil associated with the neck, wing, and halteres, and a set of intermediate neuropils including the tectulum that may coordinate the action of the legs and wings^2^. Also within the thoracic VNC are descending^3^ and ascending^4^ axons that connect the VNC and the brain. These tracts run through the neck or cervical connective, which—like the VNC—is inaccessible in most preparations.

The VNC of adult *Drosophila* is the site where some higher-order decisions are transformed into actions. Adult flies engage in complex limbed behaviors including walking^5,6^, reaching^7^, escape jumping^8^, courtship tapping^9^, aggressive boxing^10^, and grooming^11^. Our current understanding of how thoracic circuits coordinate these actions is entirely based on behavioral genetics or recordings from a few neurons in tissue explants^12^, immobilized animals^13–15^, or sharp electrode studies in larger insects^16,17^.

To fully understand how thoracic circuits orchestrate limb movements, it is necessary to record the activity of individual cells and populations of neurons during behavior. To date, these experiments have not been performed in *Drosophila* due to the difficulty of accessing the VNC in intact, behaving animals. Here we describe a preparation that overcomes this obstacle and makes it possible to record the dynamic activity of populations and sparse sets of individual neurons within adult thoracic circuits during walking, grooming, and other actions involving limb movement.

## Results

### A dissection for accessing the ventral nerve cord

**Fig. 1:**
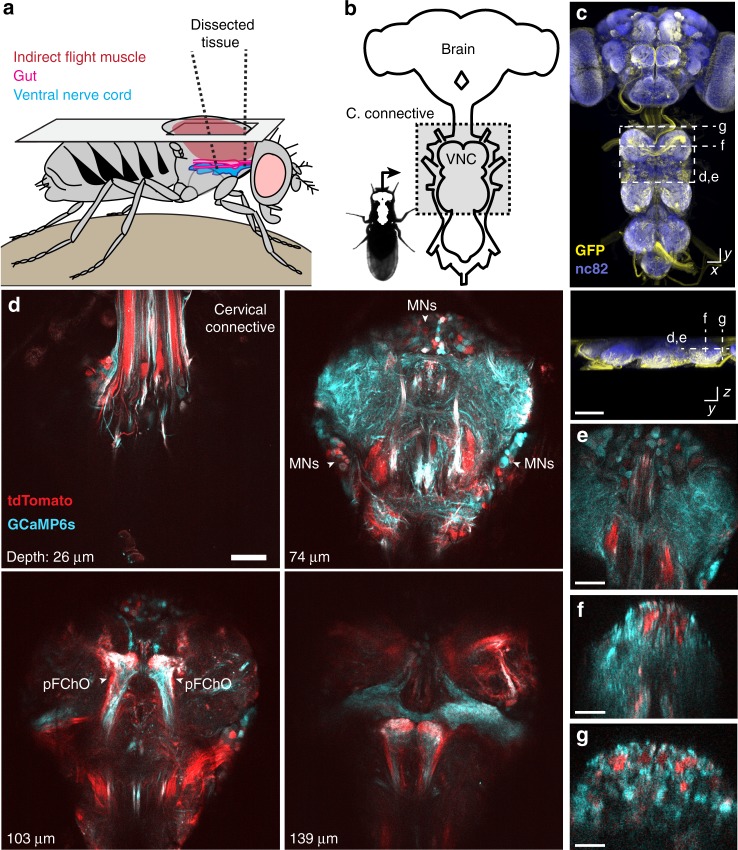
Dissection for imaging the adult *Drosophila* ventral nerve cord (VNC). **a** Schematic of the dorsal thoracic dissection. **b** Overview of newly accessible nervous tissue following thoracic dissection. **c** Confocal image of pan-neuronal driver line expression in the brain and VNC. Scale bar is 90 µm. GFP (yellow) and neuropil (nc82, blue) are labeled. Dashed lines highlight the horizontal and coronal imaging modalities used in this study. **d** Horizontal sections of the VNC imaged at different depths in an animal expressing GCaMP6s (cyan) and tdTomato (red) throughout the nervous system (*GMR57C10* *>* *GCaMP6s*; *tdTomato*). Motor neuron (MNs) cell bodies and prothoracic femoral chordotonal organ (pFChO) axon terminals are indicated (white arrowheads). Scale bar is 30 µm. **e** Horizontal section imaging of the VNC. Scale bar is 35 µm. **f** Coronal section imaging of the prothoracic neuromere. Scale bar is 50 µm. **g** Coronal section imaging of the cervical connective. Scale bar is 35 µm. Images in **e**–**g** were taken from flies expressing GCaMP6s and tdTomato throughout the nervous system (*GMR57C10* *>* *GCaMP6s; tdTomato*)

The VNC lies on the thoracic sternum—a cuticular structure that anchors the leg muscles and the proximal leg segments to the thorax (Fig. 1a). Consequently, it is difficult to access the VNC by removing ventral thoracic cuticle without destroying musculoskeletal elements required for limb movement. We chose instead to access the VNC dorsally at the expense of flight-related behaviors^18^. This approach requires removing the prescutum and scutum of the dorsal thoracic cuticle, the indirect flight muscles (IFMs), and transecting the proventriculus, crop, and salivary glands of the gut (Fig. 1a, Supplementary Fig. 1, see Methods).

Using this technique, it is possible to perform functional imaging in flies that are still capable of exhibiting robust behavior, such as walking and grooming, for up to at least 4 h. In one round of studies (*n* = 46 flies) by a newly trained experimenter 46% of animals produced behaviors, 26% had limb movement deficiencies, and 28% were incapacitated. When comparing walking behaviors between flies with or without a thoracic dissection, we found that dissected flies generate locomotor bouts with likelihoods and velocities within the range of those observed in non-dissected animals (Supplementary Fig. 2; *n* = 20 dissected and 20 non-dissected flies). We note, however, that there are more examples of highly active non-dissected animals. We also found that, on average, dissected animals generate longer bouts (Supplementary Fig. 2b; *P* < 0.05 Mann-Whitney *U*-test). This may be due to the fact that we only recorded from dissected animals that produced limb movements in response to touch or puffs of air. Therefore, they may also have been in a higher state of arousal.

Next, to illustrate the extent of optical access, we drove expression of the genetically encoded calcium indicator, GCaMP6s^19^, together with tdTomato^20^—a fluorophore that serves as an anatomical fiduciary—throughout the entire nervous system (*GMR57C10* > *GCaMP6s; tdTomato*)^21^, (Fig. 1b, d–g and Supplementary Movie 1). To perform 2-photon microscopy in semi-intact, behaving animals, we constructed a customized fly holder and spherical treadmill (Supplementary Fig. 3) that, in contrast to previous methods used to record neural activity in the brain^18,22,23^, permits optical access to the VNC along with unobstructed videography of limb movements.

### Imaging the activity of populations of neurons in the VNC

By scanning horizontal *x*–*y* image planes in animals expressing GCaMP6s and tdTomato pan-neuronally (*GMR57C10* > *GCaMP6s; tdTomato*), we could record the detailed time course of neural activity in the prothoracic neuromere during walking and grooming (Fig. 1c, e and Supplementary Movie 2). Alternatively, we could use a piezo-driven objective to scan coronal *x*–*z* image planes. These coronal sections allowed us to simultaneously record neural activity across different depths of the VNC corresponding to distinct layers housing sensory neuron axons^4^, interneurons^15^, and motor neuron dendrites^24^ (Fig. 1c, f; Supplementary Movie 3), or to monitor activity patterns across populations of descending^3,25^ and ascending fibers^4,12,25^ within the thoracic cervical connective (Fig. 1c, g and Supplementary Movie 4). Thus, we confirmed that our preparation provides optical access to previously inaccessible thoracic neural populations in behaving adult flies.

**Fig. 2:**
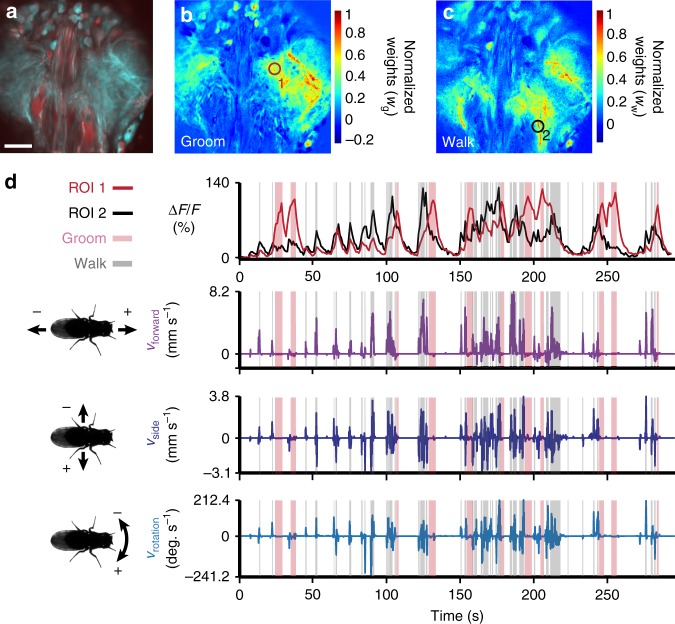
Recording populations of neurons in the VNC during behavior. **a** Standard deviation time projection for an experiment performing horizontal section imaging of the VNC. Scale bar is 35 µm. **b**, **c** Heat maps of linear regression weights *w*_g_ and *w*_w_ showing the pixel-wise relationships between fluorescence traces and **b** grooming or **c** walking, respectively. Weights are normalized to the maximum for each image. Data are from the experiment shown in panel **a**. **d** ROI-associated fluorescence traces (red from panel **b**, black from panel **c**) (top). Shaded regions indicate semi-automatically detected bouts of grooming (pink) or walking (gray). Corresponding forward, sideways, and rotational velocities of the fly (bottom)

During behavior, the VNC moves and deforms. To overcome these image analysis obstacles, we used a non-parametric, variational image registration approach, designed to model arbitrarily complex deformations (Supplementary Fig. 4 and Supplementary Movie 5, see Methods). After successful image registration, we used a semi-automated approach to annotate walking and grooming behaviors (Supplementary Movie 6, see Methods) and regressed these two datasets to identify VNC regions whose activity patterns correlated with walking and grooming (Fig. 2). We anticipate that further improvements in image registration will make it possible to build similar behavior-function maps from dense neural population imaging data in which the activity patterns of individual neurons can be identified.

### Imaging the activity of sparse sets of neurons in the VNC

Using *Drosophila*, it is possible to repeatedly and systematically investigate the functional properties of sparse sets of genetically identifiable neurons. In a recent study, a thermogenetic activation screen was used to identify a pair of descending neurons—Moonwalker Descending Neurons (MDNs)—that cause flies to walk backwards^25^. Additionally, concurrent thermogenetic activation of ascending neurons that project from the VNC to the brain—Moonwalker Ascending Neurons (MANs)—resulted in even more sustained backwards walking, perhaps by arresting forward walking^25^. Although these activation experiments show that MDNs and MANs play an important role in the control of backwards walking, their native activity patterns and the means by which they regulate or encode limb movements remain unknown.

**Fig. 3:**
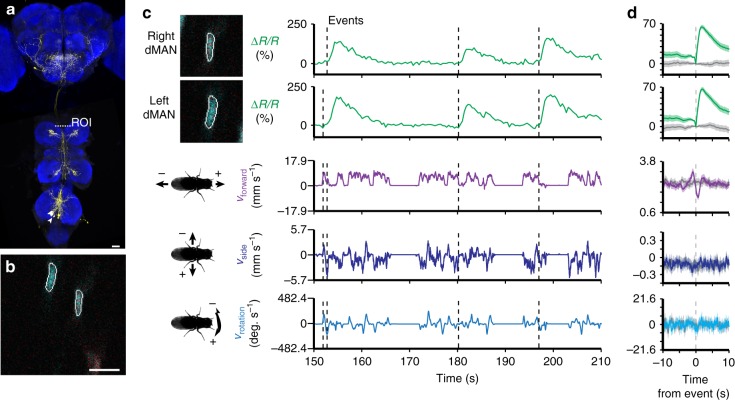
Neural activity of dMANs in the thoracic cervical connective during behavior. **a** Confocal image of *MAN-Gal4* driver line expression in the brain and VNC. Scale bar is 40 µm. Neuronal GFP (yellow) and neuropil (nc82, blue) are labeled. A dashed white line highlights the thoracic *x*–*z* plane imaged. **b** Coronal section of the thoracic cervical connective in an animal expressing GCaMP6s (cyan) and tdTomato (red) in MANs (*MAN* *>* *GCaMP6s; tdTomato*). Scale bar is 3.5 µm. **c** Separated ROIs (top-left) and associated fluorescence signals from right and left dMANs (top-right). Corresponding forward, sideways, and rotational velocities of the fly (bottom-right). Events are indicated as dashed gray lines. **d** Summary of dMAN activity and spherical treadmill rotations with respect to fluorescence events (748 left neuron and 746 right neuron events) aligned to 0 s (dashed gray line). Control data in which events are time-shuffled are overlaid in gray. Shown are the means (solid line) and bootstrapped 95% confidence intervals (transparencies)

Because MAN and MDN axons terminate in the gnathal ganglia (GNG) and the VNC—both relatively inaccessible regions of the nervous system—it is difficult to record the activity of these cells during behavior. We used our functional imaging approach to overcome this challenge and recorded the activity of ascending and descending neurons within the VNC. To overcome vertical movement artifacts associated with walking, we performed coronal section imaging of their axons within the cervical connective (e.g., Fig. 3a).

### Activity patterns of moonwalker ascending neurons

Using this approach, MAN axons are visible as small ellipses (Fig. 3b). The MAN split*-*GAL4 line we used drives expression of GCaMP6s and tdTomato (*MAN* *>* *GCaMP6s; tdTomato*) in a pair of dorsal and a pair of ventral neurons. We focused our analysis on the dorsal pair of neurons—hereafter referred to as dMANs—because they showed conspicuous changes in activity (Fig. 3c). The activity of left and right dMANs were strongly correlated (Supplementary Fig. 5a; Pearson’s *r* = 0.96 ± 0.01, *n* = 5 flies), allowing us to study their collective response properties. Specifically, we automatically identified the occurrence of transient increases in dMAN fluorescence—referred to as ‘events’—and examined corresponding behaviors reflected by rotations of the spherical treadmill (see Methods). Our analysis revealed that dMAN events were associated with rapid bimodal anterior-posterior rotations of the spherical treadmill (Fig. 3d, *n* = 748 left and 746 right dMAN events from 9773 s of data from 5 flies). Through close inspection of the video data, we observed that these rotations occur when flies extend all six legs to push down on the spherical treadmill (Supplementary Movies 7 and 8).

### Activity patterns of moonwalker descending neurons

**Fig. 4:**
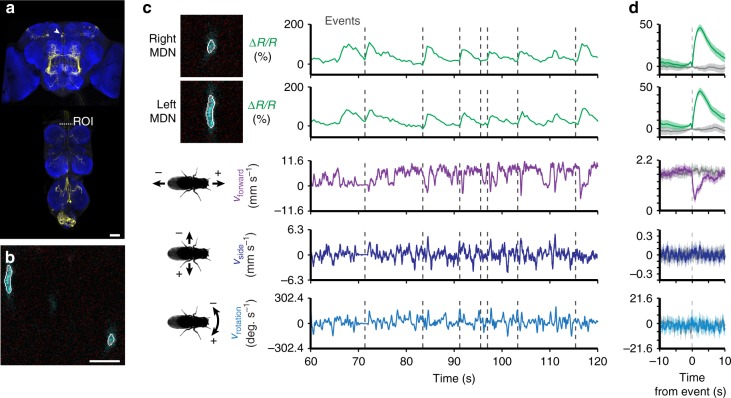
Neural activity of MDNs in the thoracic cervical connective during behavior. **a** Confocal image of *MDN-1-Gal4* driver line expression in the brain and VNC. Scale bar is 40 µm. Neuronal GFP (yellow) and neuropil (nc82, blue) are labeled. A dashed white line highlights the thoracic *x*–*z* plane imaged. **b** Coronal section of the thoracic cervical connective in an animal expressing GCaMP6s (cyan) and tdTomato (red) in Moonwalker Descending Neurons (*MDN-1* *>* *GCaMP6s; tdTomato*). Scale bar is 6 µm. **c** Separated ROIs (top-left) and associated fluorescence signals from right and left MDNs (top-right). Corresponding forward, sideways, and rotational velocities of the fly (bottom-right). Events are indicated as dashed gray lines. **d** Summary of MDN activity and spherical treadmill rotations with respect to fluorescence events (900 left neuron and 900 right neuron events) aligned to 0 s (dashed gray line). Control data in which events are time-shuffled are overlaid in gray. Shown are the means (solid line) and bootstrapped 95% confidence intervals (transparencies)

Next, we asked to what extent MDNs are active during periods of backwards walking, a possibility suggested by behavioral responses to thermogenetic^25^ and optogenetic^26^ MDN stimulation. To address this question, we performed coronal section imaging of the thoracic cervical connective in flies expressing GCaMP6s and tdTomato in MDNs (*MDN-1* *>* *GCaMP6s; tdTomato*) (Fig. 4a, b). As for dMANs, left and right MDN activity patterns were strongly correlated (Supplementary Fig. 5b; Pearson’s *r* = 0.93 ± 0.001, *n* = 3 flies), allowing us to study their collective response properties. As predicted, MDNs became active prior to anterior rotations of the spherical treadmill, corresponding to brief episodes of backward walking (Fig. 4c, d, *n* = 900 left and 900 right MDN events from 3 flies and 7790 s of data; Supplementary Movies 9 and 10).

### Activity patterns of previously uncharacterized descending neurons

**Fig. 5:**
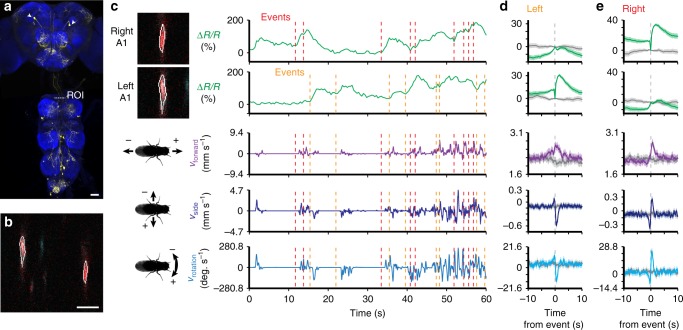
Neural activity of A1 neurons in the thoracic cervical connective during behavior. **a** Confocal image of *DNa01-Gal4* driver line expression in the brain and VNC. Scale bar is 40 µm. Neuronal GFP (yellow) and neuropil (nc82, blue) are labeled. A dashed white line highlights the thoracic *x*–*z* plane imaged. **b** Coronal section of the thoracic cervical connective in an animal expressing GCaMP6s (cyan) and tdTomato (red) in A1 neurons (*A1* *>* *GCaMP6s; tdTomato*). Scale bar is 5 µm. **c** Separated ROIs (top-left) and associated fluorescence signals from right and left A1 neurons (top-right). Corresponding forward, sideways, and rotational velocities of the fly (bottom-right). Events are indicated as dashed red and orange lines for right and left A1 neuron events, respectively. **d**, **e** Summary of A1 neural activity and spherical treadmill rotations with respect to **d** left (1644 events) or **e** right (1651 events) A1 neuron fluorescence events aligned to 0 s (dashed gray line). Control data in which events are time-shuffled are overlaid in gray. Shown are the means (solid line) and bootstrapped 95% confidence intervals (transparencies)

In addition to resolving the functional properties of previously identified neurons, our method can facilitate the discovery of novel cell classes that are active during walking, grooming, and other behaviors involving the limbs or abdomen. As a proof-of-concept, we selected four split-GAL4 lines^27^ that drive sparse expression in pairs of descending neurons^3^ whose axons project to leg neuromeres in the VNC (classes DNa01, DNb06, DNg10, and DNg13). We did not observe fluorescence responses during grooming or locomotion in DNg10, or DNg13 cells. DNb06 activity appeared to be only partially correlated with locomotion. By contrast, we observed that DNa01 neurons—hereon referred to as A1 cells—had activity patterns that were clearly linked to locomotor behaviors (*A1* *>* *GCaMP6s; tdTomato*) (Fig. 5a, b and Supplementary Movie 11). The activity of left and right A1 neurons were not highly correlated (Fig. 5c and Supplementary Fig. 5c; Pearson’s *r* = 0.53 ± 0.17, *n* = 4 flies). Therefore, we investigated the response properties of the left and right cells separately. We found that although the activities of both cells are linked to forward walking, events associated only with left A1 activity were correlated with negative medial-lateral and yaw rotations, or leftward turning by the fly (Fig. 5d and Supplementary Movie 12; *n* = 1644 events from 4 flies and 8784 s of data). As predicted from the bilateral symmetry of these cells, activity in the right A1 neuron coincided with positive medial-lateral and yaw rotations, or rightward turning (Fig. 5e and Supplementary Movie 13; *n* = 1651 events from 4 flies and 8784 s of data).

### Facilitating access to the VNC by inducing IFM cell death

**Fig. 6:**
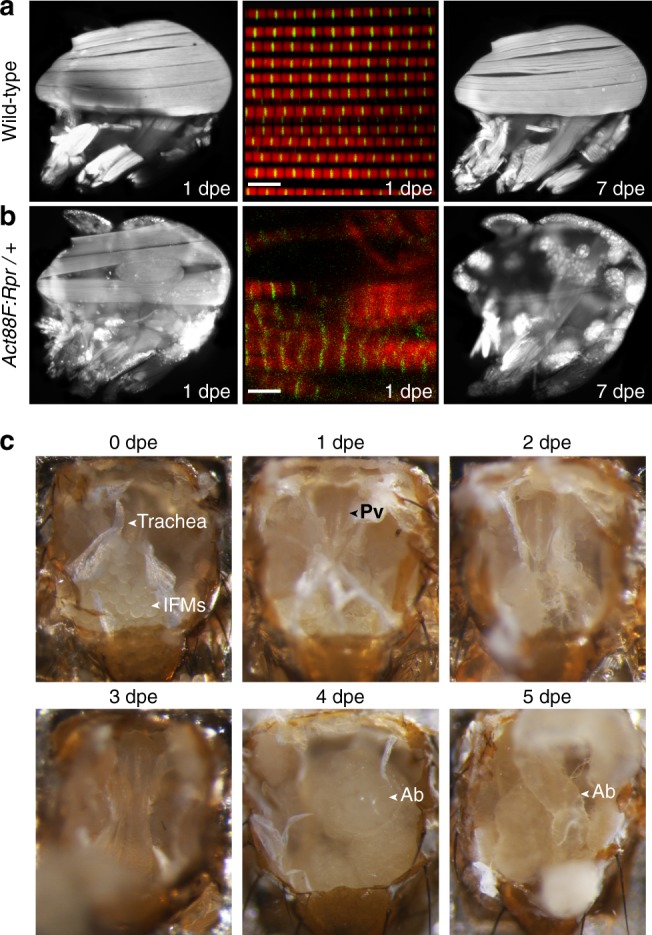
Indirect flight muscle degradation in *Act88F:Rpr* animals. Confocal images of dorsal longitudinal IFMs (DLMs) stained with TRITC-Phalloidin at 1 dpe (left), 7 dpe (right), or whole-mount confocal micrographs of myofibrillar structure (middle) for **a** wild-type, or **b** *Act88F:Rpr* heterozygous flies. Scale bars are 5 μm. **c** IFM degradation over time in heterozygous *Act88F:Rpr* animals (*Act88F:Rpr/elav-Gal4; UAS-GCaMP6s/+*). IFMs are already absent at 1 dpe, revealing the underlying proventriculus (Pv). At 4 dpe and later, abdominal gut (Ab) invades the thoracic cavity

Our approach for recording neural activity in the VNC of behaving *Drosophila* opens up many new avenues for studying premotor and motor circuits. Nevertheless, we can envision further improvements that will accelerate the study of the thoracic nervous system. For example, in our preparation we found it challenging and time-consuming to remove indirect flight muscles (IFMs) that fill most of the thorax. Although large, these muscles are quite fragile and tend to disintegrate over several hours after the cuticle of the notum is removed. However, to increase the speed and efficiency of our dissection, we devised a transgenic strategy to selectively ablate IFMs. We drove the expression of Reaper—a protein involved in apoptosis^28^—in IFMs using a 5′ *Act88F* promotor sequence^29^. This loss results in highly elevated or slightly depressed wings—phenotypes seen in IFM developmental mutants^30^. *Act88F:Rpr* animals show a nearly complete loss of IFMs after 7 days post-eclosion (dpe) when raised at 25 °C (Fig. 6a, b). The heterozygous *Act88F:Rpr* transgenic background greatly accelerated the dorsal thoracic dissection. Immediately following eclosion (0 dpe), we observed prominent degradation of IFMs (Fig. 6c). For imaging, *Act88F:Rpr* was most effective at up to 3 dpe: after this stage, the abdominal gut often entered the thoracic cavity. *Act88F:Rpr* also increased the success of dissections: in one round of studies (*n* = 15 flies) 73% of animals produced behaviors, only 13% had limb movement deficiencies, and only 13% were incapacitated.

We next assessed the degree to which *Act88F:Rpr* might negatively impact tissues beyond the IFMs, including neurons and muscles. Due to the difficulty of identifying regions with Rpr-driven cell death, we instead measured fluorescence in transgenic animals expressing GFP driven by the same promoter sequence (*Act88F:eGFP*)^29^. We observed green fluorescence very rarely and at very low levels outside—but not within—the central nervous system (Supplementary Fig. 6). Unlike a previous report^31^, we also did not observe any fluorescence in the leg muscles of *Act88F:eGFP* animals (Supplementary Fig. 7). This difference may be due to the use of different insertion sites. These anatomical observations were further supported by behavioral responses to antennal infrared laser stimulation: *Act88F:Rpr* (*Act88F:Rpr; UAS-GCaMP6s-p2A-tdTomato; GMR57C10-GAL4*) and control (*+;UAS-GCaMP6s-p2A-tdTomato; GMR57C10-GAL4*) animals exhibited qualitatively indistinguishable walking behaviors at 7 dpe. However, we did observe very small, quantitative differences in walking speed near the end of the stimulation pulse (Supplementary Fig. 8, *n* = 15 *Act88F:Rpr* animals and *n* = 15 control animals; *n* = 10 responses per animal; *P* < 0.001 Friedman test, then *P* < 0.05 Mann–Whitney *U*-test with Holm-Bonferroni correction). Finally, we recorded neural activity in *Act88F:Rpr* flies and observed no qualitative differences between these animals (*Act88F:Rpr; elav-GAL4/+; UAS-GCaMP6s/+*) and their control counterparts (*elav-GAL4/+; UAS-GCaMP6s/+*) (Supplementary Movie 14).

## Discussion

Several additional modifications might increase the power of our VNC imaging approach. First, we used coronal section imaging to record from sparse sets of descending and ascending neurons. This strategy was chosen to overcome movement issues observed during horizontal section imaging (Supplementary Movie 15). Technologies for reducing axial resolution to achieve video-rate 2-photon imaging could be used to overcome this problem^32^. Second, we currently resect the gut to gain access to the VNC. This intervention does not profoundly impact limb movements—we demonstrate robust locomotor behaviors across 40 minutes (Supplementary Movies 16–18)—but we predict that efforts to leave the gut intact will permit even longer recordings.

We have shown that we can record the activity of large VNC neural populations (Fig. 2), as well as sparse cell classes with known (Figs. 3 and 4), or unexplored functional properties (Fig. 5). Our findings have been confirmatory—MDN activity correlates with backward walking—as well as unexpected—dMAN activity correlates with limb pushdown behaviors. With fluorescence decay transients (*t*_1/2_) on the order of 1s^19^, we cannot currently establish whether these signals precede these behaviors. However, based on previous observations^25^, MDNs and perhaps A1 descending neurons likely drive locomotor behaviors. By contrast, MAN activity may report behavioral signals to higher-order decision-making centers in the brain. These first results suggest that our recording method, in conjunction with genetic behavioral screens^25,33,34^, will become an indispensable tool for unraveling how signaling from the brain and neural dynamics within the VNC give rise to complex motor actions.

## Methods

### *Drosophila* lines

Several lines (*GMR57C10-Gal4*, *elav-Gal4, UAS-GCaMP6s*, *UAS-GCaMP6f*, *UAS-CD4:tdGFP*, and *UAS-tdTomato)* were obtained from the Bloomington Stock Center. *MAN-Gal4* (*VT50660-AD; VT14014-DBD*) and *MDN-1-Gal4* (*VT44845-DBD; VT50660-AD*) were provided by B. Dickson (Janelia Research Campus). *DNa01-Gal4* (SS00731: *GMR22C05-AD; GMR56G08-DBD*), *DNb06-Gal4* (SS02631: *GMR20C04-AD; BJD113E03-DBD*), *DNg13-Gal4* (SS02538: *BJD118A10-AD; BJD123E03-DBD*), and *DNg16-Gal4* (SS01543: *BJD104A07-AD; BJD107F12-DBD*) were provided by G. Rubin (Janelia Research Campus).

### Generation of Act88F:Rpr construct and flies

*Actin88F:Rpr* strains (*Act88F:Rpr* flies) were generated using an *Actin88F:eGFP* construct^29^. The *Act88F:GFP* line, which houses an *eGFP* construct driven by a 2053 bp region of the *actin88F* promoter, was obtained from R. Benton (University of Lausanne). An *Act88F:Rpr* construct was generated by first using the following primer pair, to add a KpnI restriction site to the 5′ end of a *rpr* cDNA clone (IP02530, *Drosophila* Genomics Resource Center, Bloomington, IN) and an XbaI site to the 3′ end of the open reading frame, via a QuikChange Site-directed mutagenesis kit (Agilent Technologies):

Forward primer 5′ AGACGGTACCATGGCAGTGGCATTC 3′

Reverse primer 5′ GCCGCGTCTAGATCATTGCGATGGCTT 3′

The *Rpr* construct was then spliced into the *Act88F:eGFP* construct behind the *Act88F* promoter in place of the *eGFP* sequence. The *Act88F:Rpr* construct was injected into attp18 *Drosophila* embryos for PhiC31 integrase-mediated site-specific transgenesis^35^ (transgene landing site cytolocation 6C12) by BestGene Inc. (Chino Hills, CA). For some experiments, this transgene was combined with *UAS-GCaMP6s-p2A-tdTomato* (generated in the laboratory of M.H.D.).

### Fluorescence imaging of indirect flight muscles

Fluorescent microscopy of hemi-thoraces was performed^36,37^. Briefly, flies were anesthetized and their heads and abdomens were then removed. Thoraces were fixed overnight in 4% paraformaldehyde at 4 °C and rinsed in 1× phosphate buffered saline (PBS) the following day. The specimens were arranged on a glass slide, snap frozen in liquid nitrogen and bisected down the midsagittal plane using a razor blade. IFMs were stained with Alexa-Fluor 568 Phalloidin (1:100 in PBS with 0.1% Triton-X (PBST)) overnight at 4 °C, rinsed with PBS and visualized using EVOS® FL Cell Imaging System (Life Technologies) at ×4 magnification. For whole-mount imaging of IFM myofibrils, flies were prepared and thoraces bisected as described above. Hemi-thoraces were stained with Alexa-Fluor 568 Phalloidin (1:100 in PBST) overnight at 4 °C. Samples were rinsed in PBS, mounted with Vectashield (Vector Laboratories) and visualized using a Leica TCS SPE RGBV confocal microscope (Leica Microsystems) at 100x magnification.

### Immunofluorescence imaging of brains and ventral nerve cords

Brains and VNCs were dissected out of 2–3 dpe female flies in PBS. Tissues were then fixed for 20 min in 4% paraformaldehyde in PBS at room temperature. After fixation, brains and VNCs were washed 2–3 times in PBS with 1% Triton-X-100 (PBST) for 10 min each and then incubated at 4 °C overnight in PBST. Samples were then placed in PBST with 5% normal goat serum (PBSTS) for 20 min at room temperature. They were then incubated with primary antibodies (rabbit anti-GFP at 1:500, Thermofisher RRID: AB_2536526; mouse anti-Bruchpilot/nc82 at 1:20, Developmental Studies Hybridoma Bank RRID: AB_2314866) diluted in PBSTS for 48 h at 4 °C. Brains and VNCs were rinsed 2–3 times in PBST for 10 min each before incubation with secondary antibodies (goat anti-rabbit secondary antibody conjugated with Alexa 488 at 1:500; Thermofisher; goat anti-mouse secondary antibody conjugated with Alexa 633 at 1:500; Thermofisher) diluted in PBSTS for 48 h at 4 °C. Finally, brains and VNCs were rinsed 2–3 times for 10 min each in PBST and mounted onto slides with bridge coverslips in Slowfade mounting-media (Thermofisher).

Samples were imaged using a Carl Zeiss LSM 700 Laser Scanning Confocal Microscope with the following settings: ×20 magnification, 8-bit dynamic range, 2× image averaging, 0.52 × 0.52 μm pixel size, 0.57 μm *z*-step interval. Standard deviation z-projections of imaging volumes were made using Fiji^38^. To compare GFP expression in the central nervous system, laser intensity and PMT gains for the green channel were kept constant across wild-type, *A1* > *GFP*, and *Act88F:GFP* samples.

### Imaging GFP expression in leg muscles

Legs were manually dissected at the body-coxa joint and mounted onto glass slides using double-sided tape. Slowfade mounting-media (Thermofisher) was then added to the space between the cover slip and the slide. We then recorded GFP fluorescence in the green channel using an LSM 700 Laser Scanning Confocal Microscope (Zeiss). Laser intensity, PMT gains, and scanning parameters were kept constant across wild-type, *MHC* *>* *GFP* and *Act88F:GFP* animals: ×20 magnification, 8-bit dynamic range, ×8 image averaging, 0.63 × 0.63 µm pixel size, and 10 µm *z*-step interval. Cuticular auto-fluorescence was also recorded in the red channel.

### Thoracic dissection for VNC imaging

Custom holders used to mount flies during imaging were fabricated as described previously^39^. For VNC imaging, these stages were modified to have (i) flat rather than folded steel shims, and (ii) chamfered vertices to make the spherical treadmill visible to optic flow sensors (Shapeways, ‘file’). Steel shims were fabricated from 0.001” Stainless Steel, type 316 soft annealed (McMaster-Carr, part #2317K11). Shims were etched (Etchit, Buffalo, MN) to generate rectangular holes as explained ‘here’. The shim design file can be found ‘here’.

All experiments were performed on 1–3 dpe female flies raised at 25 °C on standard cornmeal food on a 12 h light:12 h dark cycle. Flies were anesthetized at 4 °C. A female fly was selected and, in some cases, its wings were clipped to simplify the mounting process. The fly’s dorsal thorax was then pushed through a hole in the steel shim of the imaging stage. The stage was then flipped over, UV-curing glue (Bondic, Aurora, ON Canada) was carefully applied around the perimeter of the thorax and cured by UV illumination (LED-200, Electro-Lite Co. Bethel, CT USA). UV glue was then used to fix the head and abdomen to the underside of the stage. The stage was then filled with extracellular saline^18^. Under a high-magnification dissection microscope (Leica M165C), a hypodermic needle (30 G, BD PrecisionGlide, Franklin Lakes, NJ USA) was used to slice and lift the cuticle off the dorsal thorax^40^, being careful not to sever the neck connective. Subsequently, in non-*Act88F:Rpr* animals, a pair of dull forceps was used to remove IFMs, predominantly from the anterior-medial region of the thorax overlying the gut (this step is unnecessary in *Act88F:Rpr* animals). This process exposes the dorsal surface of the proventriculus—a large bulbous gut structure. With great care, a pair of super-fine forceps was then used to grasp and lift the proventriculus to displace much of the gut (including the crop and salivary glands) from the more ventrally located nervous tissue. With the gut thus elevated, ultra-fine clipper scissors (Fine Science Tools, Foster City, CA USA) were used to transect it at its anterior-most section. The proventriculus was then peeled back and a posterior incision was made to completely remove these portions of the gut, revealing the underlying nervous tissue. Notably, this dissection also removes the aorta, restricting hemolymph flow from the abdominal dorsal vessel. Nevertheless, we found that flies were viable and behaved for up to 4 h. In some cases, we observed that gut or muscle tissue would begin to obscure the VNC during imaging. Therefore, loose tissue should be removed at this stage while taking great care not to sever the VNC. After each dissection, we examined the extent to which the animal moved its legs in response to a puff of air or grabbed an object with each of its legs. This proved to be an accurate predictor of the success of the preparation. To evaluate the quality of a dissection, we examined the movements of each leg on the spherical treadmill. If a fly could walk in a coordinated manner, the dissection was considered successful. Otherwise, the animal was categorized as having a limb movement deficiency. Animals with multiple dysfunctional legs were categorized as incapacitated.

### 2-photon microscopy during behavior

Experiments were performed in the evening Zeitgeber time (Z.T.) and animals were typically imaged 30–60 min following dissection. Fly holders were secured to a raised platform over the spherical treadmill (Supplementary Fig. 3a). The VNC was then located using microscope oculars and positioned in the center of the field-of-view by 2-photon imaging.

The spherical treadmill is an aluminum rod with a ball-shaped hole milled at one end^22^. We fabricated 10 mm diameter foam balls (Last-A-Foam FR-7106, General Plastics, Burlington Way, WA USA) and manually spotted them using a Rapidograph pen (Koh-I-Noor, Leeds, MA USA) to provide high-contrast features for optic flow measurements. A 500–600 mL min^−1^ stream of filtered and humidified air was passed through the holder using a digital flow controller (Sierra Instruments, Monterey, CA USA). Movements of the ball were measured using two optical flow sensors (ADNS3080) outfitted with zoom lenses (Computar MLM3X-MP, Cary, NC USA). The ball and fly were illuminated using a pair of IR LEDs (850-nm peak wavelength) coupled to optic fibers and collimator lenses (ThorLabs, Newton, NJ USA). Optic flow measurements were passed to a microcontroller board (Arduino Mega2560) to be recorded using custom Python code. Simultaneously, video recordings of animals behaving on the ball were made using an IR-sensitive firewire camera (Basler, Ahrensburg, Germany) at approximately 30 fps.

We performed 2-photon microscopy using a Bergamo II microscope (ThorLabs) outfitted with two GaAsP PMT detectors for GCaMP6 and tdTomato imaging and coupled to a Ti:Sapphire laser (MaiTai DeepSee, Newport Spectra-Physics, Santa Clara, CA USA) tuned to 930 nm. We used an Olympus 20× water-immersion objective lens with 1.0 NA (Olympus, Center Valley, PA USA). The microscope was controlled using ThorImage software (ThorLabs). Coronal section imaging experiments were performed in Galvo-Galvo imaging mode at 6–9 Hz. This framerate varied with image size which ranged between 26.58 × 26.58 µm and 53.15 × 53.15 µm. Laser power ranged between 3 mW and 5.7 mW. Volumetric imaging is also possible with appropriate hardware (e.g., Galvo-Resonance scanner and Piezo-driven objective collar).

Occasionally, a puff of air was used to elicit walking behaviors. These puffs were digitally encoded (Honeywell AWM 3300 V, Morris Plains, NJ USA). Custom ROS software interfaced through an analog output device (Phidgets, Calgary, Canada) to ThorSync software (ThorLabs) was used to synchronize optic flow measurements, behavior videography, air puff measurements, and 2-photon image acquisition. For coronal section imaging, a Piezo collar (Physik Instrumente, Karlsruhe, Germany) was used to control rapid z-axis movements of the microscope objective lens.

To compare neural activity between control and *Act88F:Rpr* animals, we acquired 512 × 512 pixel images at 1.7 fps using a constant laser intensity and PMT gain. Selected imaging regions were empirically chosen as horizontal sections consisting of landmarks observed at ~61–65 µm depth in Supplementary Movie 1.

### Comparing walking with or without dissection

To evaluate the effects of dissection on locomotion, wild-type animals were subjected to the following procedure. Animals were mounted onto imaging stages and saline was added to each stage. Only a random subset of animals was dissected. Each mounted fly was then placed onto the spherical treadmill and its walking behaviors were recorded for 30 min. Optic flow was recorded as described above. To increase the likelihood of locomotion, a 500 ms pulse of 100% CO_2_ was directed at the fly’s antennae with a one min inter-pulse interval (0.05 l_n_ min^−1^ using a mass flow controller; Vögtlin Instruments, Switzerland).

### Infrared laser antennal stimulation

To compare walking behaviors between *Act88F:Rpr* and control animals, we stimulated their antennae with an 830 nm near infrared laser (Schäfter + Kirchhoff, Germany). We first anesthetized 7–8 dpe female animals at 4 °C and mounted them on imaging stages. Flies were then acclimated for 10 min. For each experiment, an animal received ten 2 s laser stimulation pulses (18.1 mW) to its right antenna at a 60 s inter-pulse interval. Control and *Act88F:Rpr* animals were tested in alternation to minimize the effects of circadian time on behavioral comparisons.

### Statistics

Sample sizes for animal experiments were chosen as follows: we performed at least three experiments to illustrate population and sparse neural recordings and performed more than ten experiments per group when performing statistical comparisons. A pre-established criteria of low signal-to-noise fluorescence signals resulted in the removal of two MDN experiments from our dataset. No randomization or blinding was used. For antennal laser stimulation, data were not normally distributed, thus Friedman and Mann–Whitney *U*-tests were performed. Estimates of variation are presented as mean and bootstrapped 95% confidence intervals.

### Data analysis

We analyzed all data using custom Python scripts. Because the data acquisition frequency differed for optic flow, behavior videography, and 2-photon imaging we interpolated signals to match those of the highest frequency. Subsequently, optic flow data were smoothed using a running average (window = 200 ms) and then translated into rotations s^−1^ for anterior-posterior, medial-lateral, and yaw axes^22^. To make these measurements more intuitive, rotations s^−1^ were then converted into mm s^−1^ (1 rot s^−1^ = 31.42 mm s^−1^) for anterior-posterior (*v*_forward_) and medial-lateral (*v*_side_) movements and into degrees s^−1^ (1 rot s^−1^ = 360° s^−1^) for yaw (*v*_rotation_) movements^22^.

The analysis of locomotion in dissected animals (Supplementary Fig. 2) was performed as follows*. V*_forward_ optic flow data for 20 dissected and 20 non-dissected flies were downsampled to 1500 points s^−1^ and smoothed using a running average of duration 0.2 s. To compute the percentage of time walking forward/backward, or walking sideways two thresholds, −0.31 mm s^−1^ and +0.31 mm s^−1^,were empirically defined to differentiate between standing still and forward (rightward) or backward (leftward) walking, respectively. Values above 0.31 mm s^−1^ were considered moments of forward (rightward) walking and values below −0.31 mm s^–1^ were considered moments of backward (leftward) walking. Optic flow values between these thresholds were considered moments of standing still. The percentage of time walking was calculated as the proportion of data points in which an animal was not considered standing still. Similarly, thresholds of 10.8 and −10.8 degree s^−1^ were used to defined moments of turning. A bout was defined as a continuous period of walking or turning.

Large tissue deformations could occur during behavior. Therefore, we performed post-hoc pan-neuronal image registration (Fig. 2). We registered all frames of an imaging experiment to one reference image. Because the complexity of deformations could not be captured using simple parametric motion models (e.g., affine transformations), we used a non-parametric, variational approach, designed to model arbitrarily complex deformations. We computed the motion field **w** between the reference image, denoted *I*_r_, and the image at time *t*, denoted *I*_*t*_, by solving the minimization problem

1 $$\widehat {\mathbf{w}} = {\mathrm{arg}} \mathop{\min }_{\mathbf{w}} D\left( {\mathbf{w}} \right) + \lambda \mathop {\sum }\limits_{{\mathbf{x}} \in {\mathrm{\Omega }}} \parallel \hskip -3pt \nabla {\mathbf{w}}\left( {\mathbf{x}} \right) \hskip -3pt \parallel _2^2,$$

where *D*(**w**) is a data fitting term, the second term is a regularization promoting smoothness of **w** by penalizing its gradient ∇**w**^41^, **Ω** is the discrete image domain, and the parameter *λ* balances the contributions of the two terms.

GCaMP6s images present a challenge for motion estimation because neural activity produces large local intensity changes. Therefore, we used an additional activity independent fluorophore, tdTomato, and defined a data term of the form

2 $$D\left( {\mathbf{w}} \right) = \rho \left( {{\mathbf{w}},I_{\mathrm{r}},I_t} \right) + \gamma \phi \left( {{\mathbf{w}},I_{\mathrm{r}},I_t} \right).$$

The first term models the standard assumption of conservation of intensity along the trajectory of each pixel. It is defined by

3 $$\rho \left( {{\mathbf{w}},I_{\mathrm{r}},I_t} \right) = \mathop {\sum }\limits_{{\mathbf{x}} \in {\mathbf{\Omega }}} \left| {I_t\left( {{\mathbf{x}} + {\mathbf{w}}\left( {\mathbf{x}} \right)} \right) - I_{\mathrm{r}}({\mathbf{x}})} \right|,$$

where we use an $\ell _1$ norm to gain partial robustness to intensity changes^42^. The second term in Eq. (2) is a feature matching constraint inspired by Revaud and co-workers^43^, written as

4 $$\phi \left( {{\mathbf{w}},I_{\mathrm{r}},I_t} \right) = \mathop {\sum }\limits_{{\mathbf{x}} \in {\mathbf{\Omega }}} \left\| {{\mathbf{w}}\left( {\mathbf{x}} \right) - {\mathbf{m}}({\mathbf{x}},I_{\mathrm{r}},I_t)} \right\|_1.$$

In Eqs. (2)–(4), *I*_r_ and *I*_*t*_ are from the tdTomato channel. Minimizing the function *ϕ* favors motion vectors **w**(**x**) to be close to feature correspondences **m**(**x**, *I*_r_, *I*_*t*_), computed on a sparse set of relevant keypoints. We obtain **m** with the feature matching algorithm proposed by Revaud and co-workers^43^, which is specifically designed to handle large image deformations. We compute **m** using the tdTomato imaging channel, such that the correspondences are also insensitive to the intensity changes between *I*_r_ and *I*_*t*_. As a result, the estimation is guided by reliable feature matches. The parameter *γ* balances the two terms in Eq. (2).

For each experiment, we optimized the values for *λ* and *γ* using a grid search to register horizontal section images of the VNC (Supplementary Fig. 4). As an objective function for optimization, we used the gradient of the temporal mean image^44^. Small values of *λ* (i.e., *λ* < 1000), occasionally led to artifacts in the registered images. These artifacts were associated with strong convergence in the vector field **w**(**x**) (Supplementary Fig. 4c). Therefore, we empirically defined artifacts as clusters of pixels with ${\mathrm{div}}\,{\mathbf{w}}\left( {\mathbf{x}} \right) < - 1.2$ and cardinality >20 (we obtained similar results with cardinality >5). Finally, we selected *λ* and *γ* values as those with no artifacts and the highest gradient of the mean image. Sample unregistered images, transformation vector fields, and registered images of the three optimized examples are shown in Supplementary Movie 5.

We solved the optimization problem in Eq. (1) with an alternated direction method of multiplier (ADMM) algorithm^45^. We introduced two splitting variables, associated with the regularization and the feature matching terms, respectively. Each sub-problem of the algorithm was solved analytically. We used parts of the inverse problems library described in ref. ^46^. A post processing based on weighted median filtering was applied using the method from^47^.

In Fig. 2, behaviors were semi-automatically annotated, using a custom Python module. This module allows the user to select two regions-of-interest (ROIs) on the video’s first frame. The first ROI is used to detect walking and must be positioned over the metathoracic and mesothoracic legs. The second ROI is responsible for detecting prothoracic leg grooming and must be positioned in front of the fly. To detect motion in those regions, consecutive frames are subtracted. Resulting differential images are then median blurred (radius = 5 pixels), to reduce noise. Based on this blurred image, a threshold on the number of non-zeros pixels in each of the two ROIs is applied to extract binary sequences of grooming and walking bouts. Note that prothoracic leg movements observed during walking are ignored (i.e., grooming classification is subservient to walking classification). A hysteresis filter was then applied to low-pass filter binary behavioral sequences and to remove transitions that occur over too few frames to be biologically plausible. Example ROIs and behavioral annotations are illustrated in Supplementary Movie 6. This behavior data was used in Fig. 2 as shown in Supplementary Movie 2. It was annotated using the following parameters: threshold for walking = 400, threshold for grooming = 5, hysteresis length for walking = 8, hysteresis length for grooming = 10.

For Fig. 2b, c, we used linear regression to find regions in the VNC associated with either walking or grooming. Regressors *X*_w_ and *X*_g_ (for walking and grooming, respectively) were constructed from the two behavioral sequences, *S*_w_ and *S*_g_, using Eq. (5) by convolution with an exponentially decaying Calcium signal Impulse Response (CIR) derived from the time constant measured for GCaMP6s (*t*_½_ = 1.1448 s)^19^.

5 $$\begin{array}{l}X_{\mathrm{w}} = S_{\mathrm{w}} \otimes {\mathrm{CIR}}\\ X_{\mathrm{g}} = S_{\mathrm{g}} \otimes {\mathrm{CIR}}\end{array}$$

Target functions were pixel-wise ∆*F*/*F* traces, where ∆*F* = *F*_*t*_ – *F*. *F*_*t*_ is the fluorescence at time, *t. F* is a baseline fluorescence signal measured as the average pixel value for the first ten sequential GCaMP6s images in which no cellular activity was observed (i.e., minimal and unchanging GCaMP6s fluorescence).

The regressor weights were calculated using Eq. (6).

6 $$\begin{array}{l}w_{\mathrm{w}} = \left( {X_{\mathrm{w}}^TX_{\mathrm{w}}} \right)^{ - 1}X_{\mathrm{w}}^Ty\\ w_{\mathrm{g}} = \left( {X_{\mathrm{g}}^TX_{\mathrm{g}}} \right)^{ - 1}X_{\mathrm{g}}^Ty,\end{array}$$

where *y* is the pixel-wise ∆*F*/*F* trace.

Figure 2b, c shows heat maps of the regressor weights, *w*_w_ for walking and *w*_g_ for grooming, normalized to their respective maxima. ROI 1 was chosen as a region of the heat map with a high weight for grooming but a low weight for walking. ROI 2 was chosen as the spatial location with the highest value of *w*_w_. Each ROI encompasses a region with a 15 pixel radius.

To identify process sparse neural imaging data (Figs. 3–5), ROIs were first selected using custom Python scripts that depended on OpenCV and Numpy libraries. To do this, a reference frame was selected for which the software identified all potential ROIs. To do this, the GCaMP6s image was smoothed to reduce background noise and then an Otsu filter threshold was applied to the image. An erosion factor was then applied on all objects detected within the image. Contours of all detected objects were then presented to the user for manual selection. Once these reference ROIs were selected for left and right neurons, we used a cross-correlation-based image registration algorithm^48^ to identify the most likely left and right ROIs for each image frame based on those manually selected on the reference frame. A second script was used to manually verify automatically selected ROIs and, if incorrect, to display all potential ROIs within the frame for manual selection. If erosion values yielded malformed ROIs, another script was used to manually position elliptical ROIs of arbitrary orientation on any given frame. Finally, binary ROI images were used as an image mask to extract mean fluorescence signals from the original GCaMP6s or tdTomato images. These signals were reported as %∆*R*/*R* as in ref. ^49^ to reduce the effects of motion on our measurements. Due to the absence of stimuli, the baseline *R* was calculated as the minimum ratio of GCaMP6s/tdTomato within a 2.5 s bin.

To detect transient increases in activity, we developed an algorithm based partly on ref. ^50^. We first determined when the first derivative of the %∆*R*/*R* signal crossed a threshold, which was determined by examining all derivative values for a given neuron class (MDN, MAN, or A1). We reasoned that threshold values should be characteristic and potentially different for each type of neuron because fluorescence dynamics are related to intrinsic physiological properties that can differ across neuron classes but not across experiments for a single class. We set this threshold as the 97.5th percentile for MDNs and dMANs and the 90th percentile for A1 neurons. A lower threshold value was selected for A1 neurons because many more fluorescence transients were observed in A1 traces. These transients would have been overlooked using a 97.5th percentile threshold. To identify the onset of fluorescence increases we found the nearest preceding time point where the derivative crossed zero. This zero-crossing is considered the time-point of an ‘event’ associated with the identified fluorescence increase. Events detected close to one another with no intervening derivative zero-crossing were compressed into one event associated with the first time-point. There were ~10 separate experiments per animal. Events in the first and last 10 s of each experiment were not considered since the data presentation window encompassed 10 s before and 10 s after each event.

Because left and right MDN and dMAN activities strongly covaried (Supplementary Fig. 5), an additional step was performed for event detection: if events were detected in both left and right neurons within 2 s of one another, both events were retained; otherwise, an event identified for neuron A (e.g., left MDN) and not neuron B (e.g., right MDN) was also added to neuron B’s event library.

By contrast, left and right A1 activities did not strongly covary. Therefore, events were associated with one and not the other neuron. To accomplish this, if an event was detected for both left and right A1 neurons within a time window of 0.25 s, neither of the events were used for analysis.

%∆*R*/*R* and optic flow traces linked to each event were aligned by setting the event time points to 0 s. We then computed the mean and bootstrapped 95% confidence intervals for these aligned traces using the Python Seaborn library. Optic flow and %∆*R*/*R* measurements were downsampled to 500 values s^−1^ for this analysis. To increase clarity, %∆*R*/*R* traces were baseline-subtracted to make them zero at the time of the event in the summary panels (Figs. 3d, 4d and 5d, e). Control, shuffled data (gray traces) were computed by instead assigning random time-points in place of real, identified events. These random time points were treated as real events and their mean and bootstrapped 95% confidence intervals were computed and plotted for comparison.

Covariance analysis (Supplementary Fig. 5) was performed using a custom Python script that depended on the Matplotlib and Numpy libraries. Scatter plots were computed to compare left and right neuron %∆*R*/*R* values from all experiments for each fly separately. Pearson’s *r* values are reported as mean ± standard deviation.

Event-related behaviors (Supplementary Movies 8, 10, 12 and 13) were manually selected from automatically detected events as described above. For dMANs, events were selected from among those that maximized the difference in anterior-posterior ball rotations between 1 s before and 2 s after the event. For MDNs, events were selected from among those that minimized anterior-posterior ball rotations up to 2 s after the event. For A1 neurons, events were selected from among those that maximized the average yaw ball rotations (positive for left A1 neuron examples and negative for right A1 neuron examples) for up to 2 s after the events.

In Supplementary Fig. 8, responses to near-infrared laser stimulation were averaged across 10 trials for each animal. Optic flow was downsampled to 500 values s^−1^. Mean and 95% bootstrapped confidence intervals for optic flow traces were measured and plotted using the Python Seaborn library. The Python Scipy library was used to perform Friedman and Mann–Whitney *U*-tests.

## Electronic supplementary material

Supplementary Information

Description of Additional Supplementary Files

Supplementary Movie 1

Supplementary Movie 2

Supplementary Movie 3

Supplementary Movie 4

Supplementary Movie 5

Supplementary Movie 6

Supplementary Movie 7

Supplementary Movie 8

Supplementary Movie 9

Supplementary Movie 10

Supplementary Movie 11

Supplementary Movie 12

Supplementary Movie 13

Supplementary Movie 14

Supplementary Movie 15

Supplementary Movie 16

Supplementary Movie 17

Supplementary Movie 18

## Data Availability

The source data used for the analyses in this study are available on the Harvard Dataverse on a ‘public repository’. Analysis code and sample datasets can be found ‘here’.
