# Supplementary material for: Imaging neural activity in the ventral nerve cord of behaving adult Drosophila
Source: Nat Commun. 2018 Oct 22;9:4390. doi: 10.1038/s41467-018-06857-z (PMC6197219; doi:10.1038/s41467-018-06857-z)
Supplement: Supplementary file 1 — Supplementary Information [file 41467_2018_6857_MOESM1_ESM.pdf]

Supplementary information for

# Imaging neural activity in the ventral nerve cord of behaving adult *Drosophila*.

Chin-Lin Chen‡, Laura Hermans‡, Meera C. Viswanathan, Denis Fortun,

Florian Aymanns, Michael Unser, Anthony Cammarato, Michael H. Dickinson,

Pavan Ramdya

**Supplementary Figures:**

Supplementary Fig. 1 | Illustration of dorsal dissection to access the VNC.

Supplementary Fig. 2 | Locomotion in flies with or without thoracic dissection.

Supplementary Fig. 3 | System for VNC imaging.

Supplementary Fig. 4 | Optimization of $\lambda$ and $\gamma$ values for image registration.

Supplementary Fig. 5 | Covariance in fluorescence signals between bilateral pairs of neurons.

Supplementary Fig. 6 | GFP expression in the central nervous system.

Supplementary Fig. 7 | GFP expression in leg muscles.

Supplementary Fig. 8 | Locomotor behaviors in control and *Act88F:Rpr* flies.

## Supplementary Figures

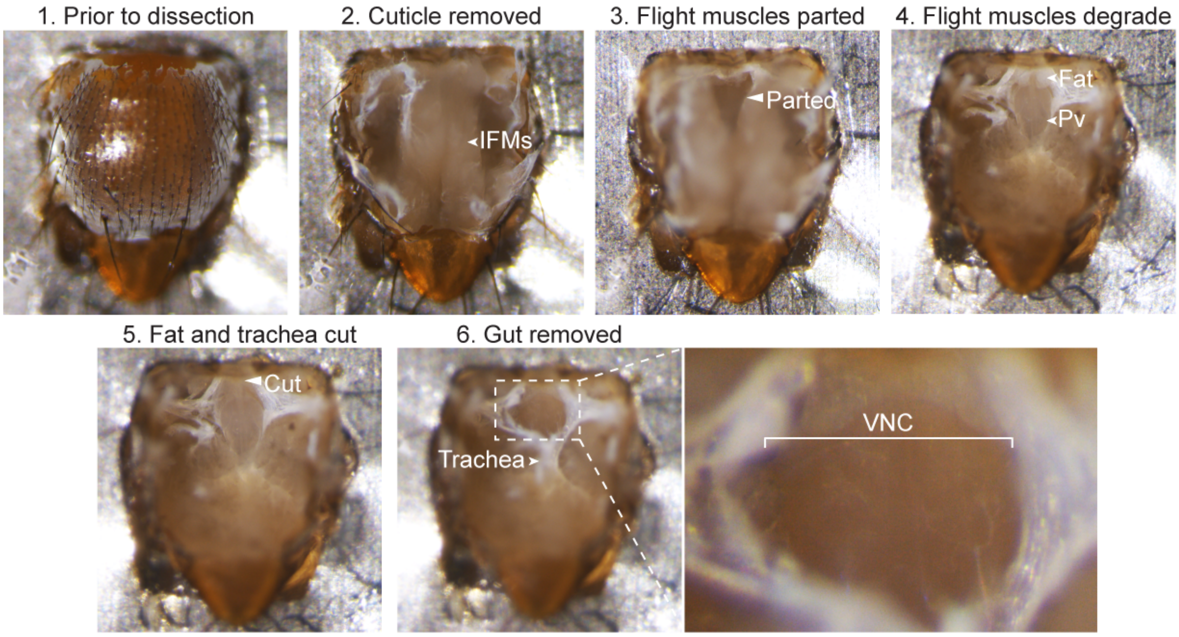


**Supplementary Figure 1 | Illustration of dorsal dissection to access the VNC.** First, the dorsal cuticle is removed, revealing indirect flight muscles (IFMs) (**steps 1-2**). The IFMs are then parted at the anterior midline (**step 3**). Over the course of ~1 h, the IFMs degrade, exposing midline trachea, fat bodies (Fat), and the proventriculus (Pv) (**step 4**). After removing these structures (**steps 5-6**), the prothoracic and mesothoracic VNC (**inset**) become accessible for 2-photon microscopy.

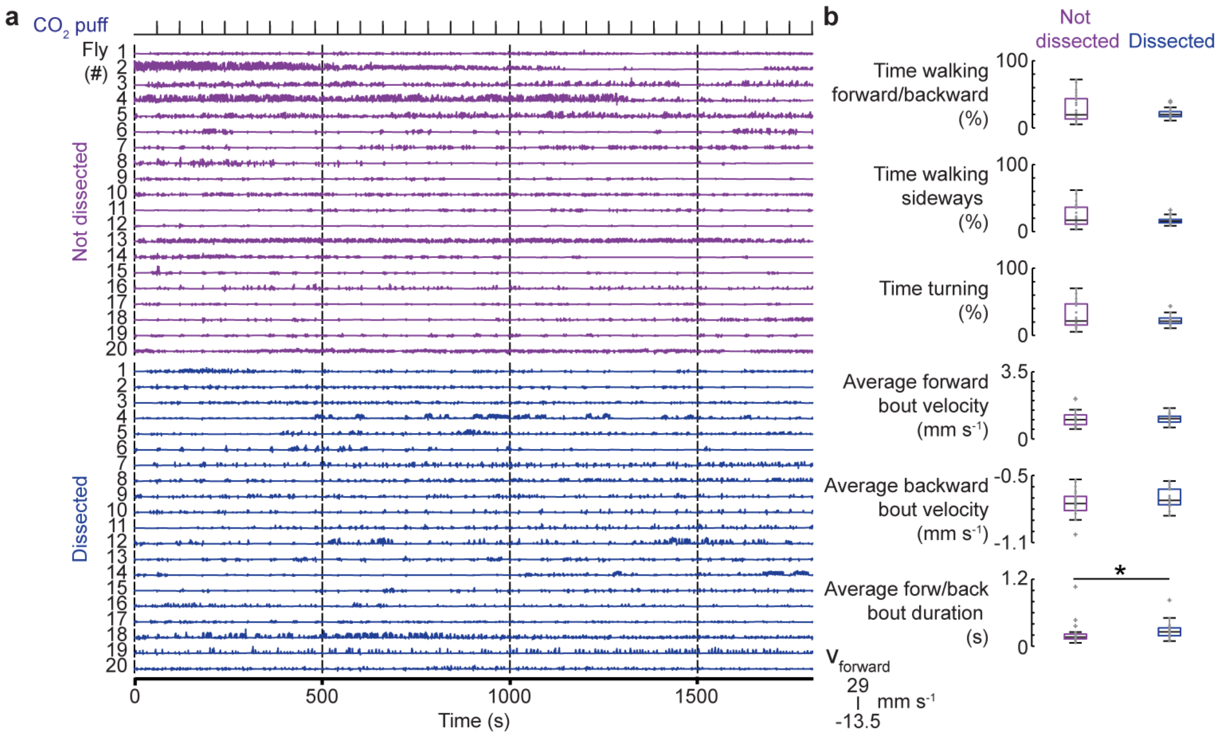


**Supplementary Figure 2 | Locomotion in flies with or without thoracic dissection. (a)** Raw spherical treadmill traces showing forward velocity for animals without **(top,** purple, *n* = *20***)**, or following a thoracic dissection that exposes the VNC **(bottom,** blue, *n* = 20**)**. Timing of $CO_2$ pulses delivered to the antennae are indicated. **(b)** Locomotor characterization of animals without **(left, purple)** or following **(right, blue)** thoracic dissection. Parameters are percent of time walking forward, walking sideways, or turning, as well as the average forward bout velocity per animal, average backward bout velocity per animal, and average forward and backward bout duration per animal. Box plots indicate median, upper, and lower quartiles. Asterisk indicates that $P<0.05$ for a Mann-Whitney *U*-test.

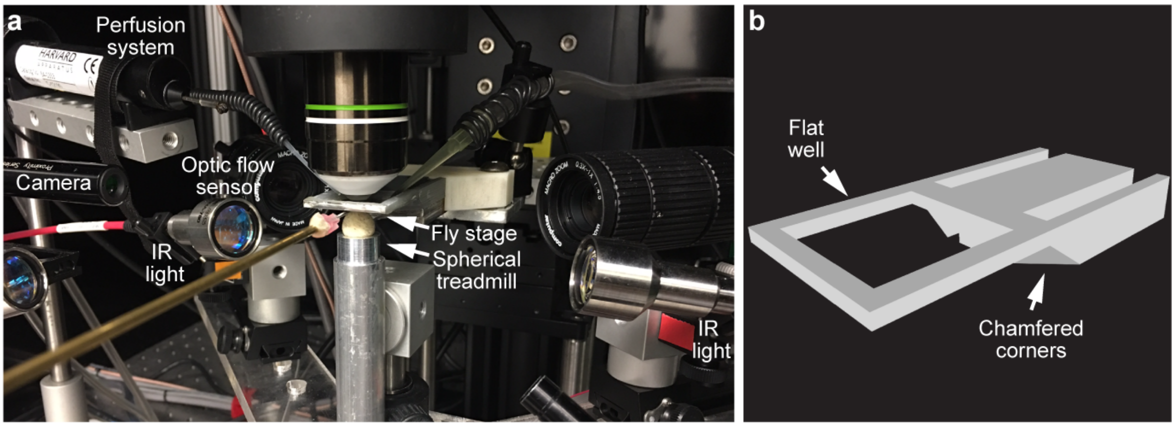


**Supplementary Figure 3** | **Experimental system for VNC imaging. (a)** Photograph of the experimental system. **(b)** A CAD schematic of the fly stage used in this study.

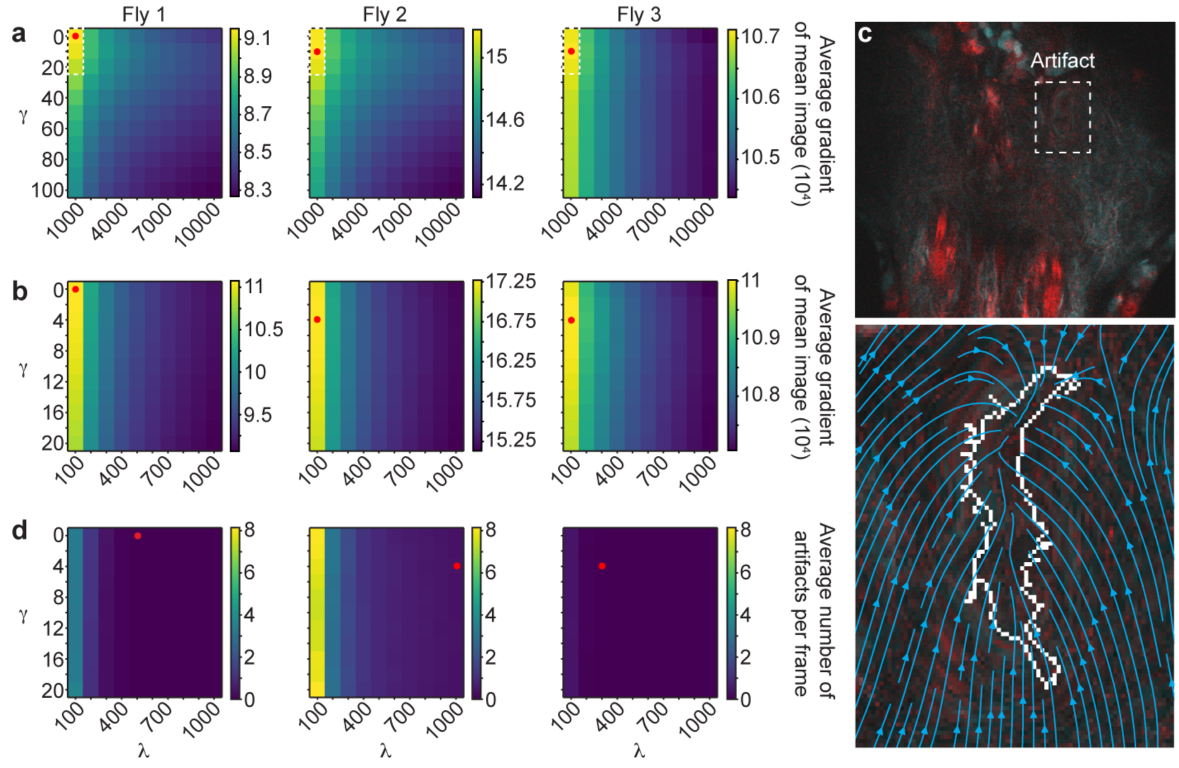


**Supplementary Figure 4 | Optimization of $\lambda$ and $\gamma$ values for image registration.** **(a-b)** The average gradient of the mean image for (**a**) a coarse grid search of $\lambda$ and $\gamma$ values for three horizontal section VNC imaging datasets ($n$ = 3 flies; same data and ordering as in **Supplementary Movie 5**). Red dots indicate the maximum value. White dashed boxes indicate the regions explored using (**b**) a finer-scale grid search. **(c)** Illustration of an image registration artifact observed for data from fly 2 when using $\lambda$ = 100 and $\gamma$ = 0 (**top**). The region in the white inset magnified and overlaid with the transformation vector field (blue arrows) and the boundary (white outline) of the artifact as determined using a threshold on divergence (**bottom**). **(d)** Average number of artifacts per frame. Red dots indicate the final $\lambda$ and $\gamma$ values used to register the data shown in **Supplementary Movie 5**. These values result in images with no artifacts while also maximizing the average gradient of the mean image.

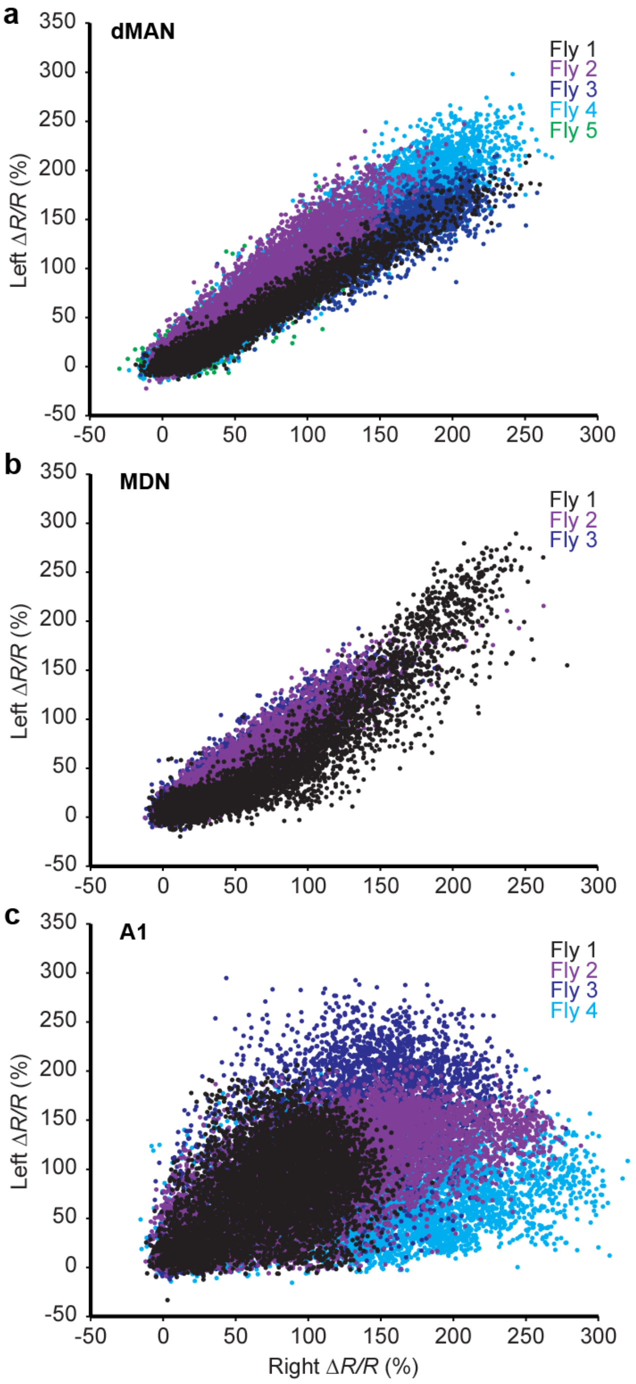


**Supplementary Figure 5 | Covariance in fluorescence signals between bilateral pairs of neurons.** Scatter plots comparing %Δ*R*/*R* signals recorded from right and left **(a)** dMAN ($n$ = 9773 s), **(b)** MDN ($n$ = 7790 s), or **(c)** A1 ($n$ = 8784 s) neuron pairs.

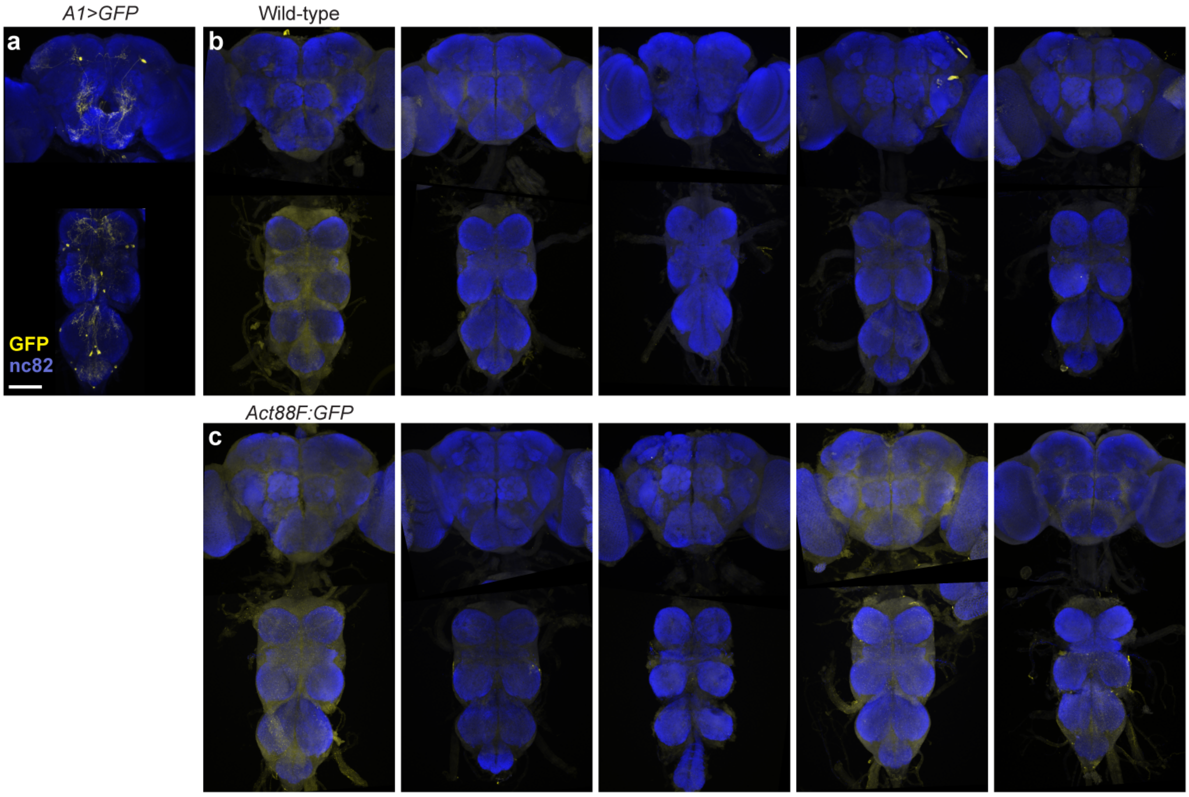


**Supplementary Figure 6 | GFP expression in the central nervous system.** Representative confocal images of brains and VNCs from **(a)** one *A1>GFP* fly: a positive control with sparse GFP expression, **(b)** five wild-type flies: negative controls without GFP expression, or **(c)** five *Act88F:GFP* flies. Immunostaining is against GFP (yellow) and nc82 (blue). Scale bar is 180 µm.

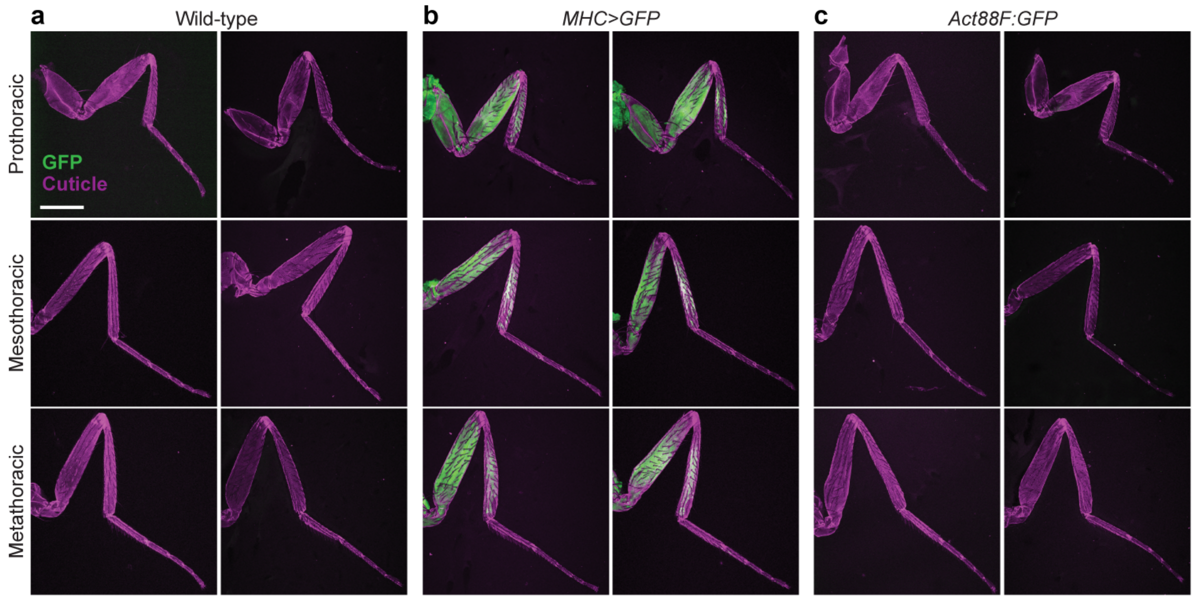


**Supplementary Figure 7 | GFP expression in leg muscles.** Representative confocal images of prothoracic, mesothoracic, or metathoracic legs from **(a)** two wild-type flies: negative controls without GFP expression, **(b)** two *MHC>GFP* flies: positive controls with leg muscle GFP expression, or **(c)** two *Act88F:GFP* flies. Shown are endogenous GFP fluorescence (green) and cuticular autofluorescence (magenta). Scale bar is 300 μm.

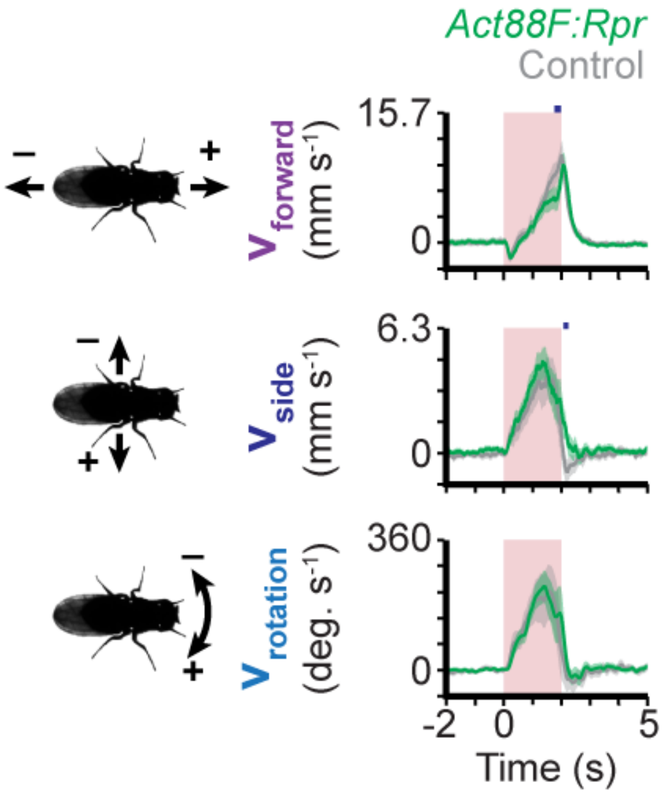


**Supplementary Figure 8 | Locomotor behaviors in control and *Act88F:Rpr* flies.** **(a)** Forward, sideways, and rotational locomotor velocities for *Act88F:Rpr* (green: *Act88F:Rpr; R57C10-Gal4 / UAS-GCaMP6s-p2A-tdTomato*), or control (grey: +; *R57C10-Gal4 / UAS-GCaMP6s-p2A-tdTomato*) animals. Shown are the means (solid lines) and bootstrapped 95% confidence intervals (transparencies) for control and *Act88F:Rpr* animals ($n$ = 10 events per fly and 15 flies per group). Red transparency indicates period of near-infrared laser stimulation of the right antenna. Blue bars show time points with significant differences between *Act88F:Rpr* and control animals ($P$<0.05; Mann-Whitney $U$-test with a Holm-Bonferroni correction).
