# Supplementary material for: Imaging neural activity in the ventral nerve cord of behaving adult Drosophila
Source: Nat Commun. 2018 Oct 22;9:4390. doi: 10.1038/s41467-018-06857-z (PMC6197219; doi:10.1038/s41467-018-06857-z)
Supplement: Supplementary file 2 — Description of Additional Supplementary Files [file 41467_2018_6857_MOESM2_ESM.pdf]

Description of Additional Supplementary Files

File Name: Supplementary Movie 1
Description: VNC imaging volume. 2-photon z-stack illustrating horizontal sections across the dorsal-ventral extent of the VNC. GCaMP6s (cyan) and tdTomato (red) are expressed throughout the nervous system (*GMR57C10>GCaMP6s; tdTomato*). Imaging depth is indicated on the top-left.

File Name: Supplementary Movie 2
Description: Horizontal VNC imaging. 2-photon imaging of a single horizontal section of the VNC in a fly that walks and grooms. GCaMP6s (cyan) and tdTomato (red) are expressed throughout the nervous system (*GMR57C10>GCaMP6s; tdTomato*). Shown are synchronized raw fluorescence images (**top-left**), %$\Delta F/F$ images (**top-right**), behavior video images (**bottom-left**), and forward, sideways, and rotational velocities of the animal (**bottom-right**). Air puff delivery is indicated by a red box above the behavior video images. Registration was performed using $\gamma = 0$ and $\lambda = 500$. Movie is 4x faster than real-time.

File Name: Supplementary Movie 3
Description: Coronal VNC imaging. 2-photon imaging of a single coronal section of the VNC in a fly that walks. GCaMP6s (cyan) and tdTomato (red) are expressed throughout the nervous system (*GMR57C10>GCaMP6s; tdTomato*). Shown are synchronized raw fluorescence images (**top-left**), %$\Delta F/F$ images (**top-right**), behavior video images (**bottom-left**), and forward, sideways, and rotational velocities of the animal (**bottom-right**). Registration was performed using $\gamma = 50$ and $\lambda = 10{,}000$. Movie is 4x faster than real-time.

File Name: Supplementary Movie 4
Description: Coronal cervical connective imaging. 2-photon imaging of a single coronal section of the cervical connective in a fly that walks. GCaMP6s (cyan) and tdTomato (red) are expressed throughout the nervous system (*GMR57C10>GCaMP6s; tdTomato*). Shown are synchronized raw fluorescence images (**top-left**), %$\Delta F/F$ images (**top-right**), behavior video images (**bottom-left**), and forward, sideways, and rotational velocities of the animal (**bottom-right**). Registration was performed using $\gamma = 50$ and $\lambda = 5{,}000$. Movie is 4x faster than real-time.

File Name: Supplementary Movie 5
Description: Motion and deformation correction. 2-photon imaging of a single horizontal section of the VNC in three different animals during behavior. Data are from Supplementary Fig. 4 (Fly 1: $\lambda = 500$ and $\gamma = 0$; Fly 2: $\lambda = 1000$ and $\gamma = 4$; Fly 3: $\lambda = 300$ and $\gamma = 4$). (**top**) Raw, unregistered, fluorescence images. (**middle**) Spatially down sampled transformation vector field used to register raw images. (**bottom**) Registered fluorescence images. Images for flies 1 and 2 are 512 x 512 pixels. Images for fly 3 are 256 x 256 pixels.

File Name: Supplementary Movie 6
Description: Semi-automated behavioral classification. Illustration of semi-automated grooming and walking annotations used for **Figure 2**. Grooming and walking ROIs are shown (white boxes). A behavioral descriptor is indicated on the top-left.

File Name: Supplementary Movie 7
Description: Coronal cervical connective imaging of dorsal Moonwalker Ascending Neurons. 2-photon imaging of a single coronal section of the cervical connective in a behaving fly. GCaMP6s (cyan) and tdTomato (red) are expressed in MANs (*MAN>GCaMP6s; tdTomato*). Raw fluorescence images of the left and right dMANs are presented and outlined by ROIs (**top-left**). These images are used to calculate %Δ*R/R* traces for each neuron (**top-right**). Corresponding behavior videography (**bottom-left**) and forward, sideways, and rotational velocities of the animal (**bottom-right**) are shown.

File Name: Supplementary Movie 8
Description: Behavioral responses associated with dorsal Moonwalker Ascending Neuron activity events. Three example behaviors (rows) produced at the onset of dMAN fluorescence events for three flies (columns). Red squares indicate the onset time of each fluorescence event (t = 0 s). Movie is 3x slower than real-time.

File Name: Supplementary Movie 9
Description: Coronal cervical connective imaging of Moonwalker Descending Neurons. 2-photon imaging of a single coronal section of the cervical connective in a behaving fly. GCaMP6s (cyan) and tdTomato (red) are expressed in MDNs (*MDN-1>GCaMP6s; tdTomato*). Raw fluorescence images of the left and right MDNs are presented and outlined by ROIs (**top-left**). These images are used to calculate % Δ*R/R* traces for each neuron (**top-right**). Corresponding behavior videography (**bottom-left**) and forward, sideways, and rotational velocities of the animal (**bottom-right**) are shown.

File Name: Supplementary Movie 10
Description: Behavioral responses associated with Moonwalker Descending Neuron activity events. Three example behaviors (rows) produced at the onset of MDN fluorescence events for three flies (columns). Red squares indicate the onset time of each fluorescence event (*t* = 0 s). Movie is 3x slower than real-time.

File Name: Supplementary Movie 11
Description: Coronal cervical connective imaging of A1 neurons. 2-photon imaging of a single coronal section of the cervical connective in a behaving fly. GCaMP6s (cyan) and tdTomato (red) are expressed in A1 neurons (*A1>GCaMP6s; tdTomato*). Raw fluorescence images of the left and right A1 neurons are presented and outlined by ROIs (**top-left**). These images are used to calculate %Δ*R/R* traces for each neuron (**top-right**). Corresponding behavior videography (**bottom-left**) and forward, sideways, and rotational velocities of the animal (**bottom-right**) are shown.

File Name: Supplementary Movie 12
Description: Behavioral responses associated with left A1 neuron activity events. Three example behaviors (rows) produced at the onset of A1 fluorescence events for three flies (columns). Red squares indicate the onset time of each fluorescence event (*t* = 0 s). Movie is 3x slower than real-time.

File Name: Supplementary Movie 13
Description: Behavioral responses associated with right A1 neuron activity events. Three example behaviors (rows) for each of three flies (columns) generated at the onset of right A1 neuron fluorescence events. Red squares indicate the time of each fluorescence event (t = 0s). Movie is 3x slower than real-time.

File Name: Supplementary Movie 14
Description: Behaviors and neural activity in *Act88F:Rpr* and control animals. Horizontal section imaging data from the prothoracic neuromere of *Act88F:Rpr* animals (**left**, *Act88F:Rpr/elav-GAL4; GCaMP6s/+;*), or control animals (**right**, *+/elav-GAL4; GCaMP6s/+;*). 2-photon imaging data are not registered.

File Name: Supplementary Movie 15
Description: Coronal and horizontal section imaging of A1 neurons in the VNC. A comparison of coronal (left), or horizontal (right) section imaging of the cervical connective in the same animal. GCaMP6s (cyan) and tdTomato (red) are expressed in A1 neurons (*A1>GCaMP6s; tdTomato*). Raw fluorescence images (**top**) are presented alongside corresponding behavior video images (**bottom**).

File Name: Supplementary Movie 16
Description: Long-term behavior in animals used to image dMAN activity. Behavior movies for three flies (*MAN>GCaMP6s; tdTomato*) during the first imaging experiment (0 min) as well as 20 min and 40 min afterwards.

File Name: Supplementary Movie 17
Description: Long-term behavior in animals used to image MDN activity. Behavior movies for three flies (*MDN>GCaMP6s; tdTomato*) during the first imaging experiment (0 min) as well as 20 min and 40 min afterwards.

File Name: Supplementary Movie 18
Description: Long-term behavior in animals used to image A1 activity. Behavior movies for three flies (*A1>GCaMP6s; tdTomato*) during the first imaging experiment (0 min) as well as 20 min and 40 min afterwards.
